# List of Accidents Admitted into the Pennsylvania Hospital, from March 21st, to April 4th, 1838

**Published:** 1838-04-11

**Authors:** 


*List of Accidents admitted into the Pennsylvania Hospital, from March 21st, to April 4th,* 1838.

One compound fracture of the fore-arm, from an injury by machinery: died in two days from mania-a-potu. One case of frost-bite in the fingers and toes: dressed with poultices; full diet and quinine internally; fingers doing well, toes mortified and separating at the second joint. One case of fractured fibula two inches above the ankle joint: dressed with the fracture box; no deformity. One case of extensive lacerated wound of the lower lip: dressed with sutures; union in five days. One case of lacerated wound of the upper lip: the edges were trimmed smooth and brought together with the hare lip suture, and the patient left the house. One case of contusion of the feet in a child, caused by a heavy weight falling on them; dressed with a fracture box and leadwater; since cured. One case of gunshot wound of the arm in a boy, caused by a marble, shot from a gun by a companion. It penetrated the arm a little below the outer condyle, and passed round the humerus to the back part of the arm, above the olecranon, where it was cut out; severe inflammation followed: the arm was placed in a carved angular splint, and poulticed; V. S. at night, ad ßℨx. a purge and low diet, with anodynes; inflammation nearly gone, wound suppurating—doing well, little constitutional irritation. One case of fracture of the femur, close to the trochanter, dressed with Physick's modification of Desault's splints.

The lacerated wound of the scalp, mentioned in the last, has nearly healed. The wounded iris, from a blast, has been discharged cured, the sight of the eye destroyed. The sprained ankle discharged, cured in two weeks. The sprain of the wrist and lacerated wound of the hand are still under treatment. The tumour of the breast, reported at length in the last is nearly well, the sutures were removed in eight days, union almost perfect.

